# Pyocin efficacy in a murine model of *Pseudomonas aeruginosa* sepsis

**DOI:** 10.1093/jac/dkab199

**Published:** 2021-06-18

**Authors:** Anne Six, Khedidja Mosbahi, Madhuri Barge, Colin Kleanthous, Thomas Evans, Daniel Walker

**Affiliations:** 1 Institute of Infection, Immunity and Inflammation, University of Glasgow, Sir Graeme Davis Building, University Place, Glasgow, G12 8TA, UK; 2 Department of Biochemistry, University of Oxford, South Parks Road, Oxford, OX1 3QU, UK

## Abstract

**Background:**

Bloodstream infections with antibiotic-resistant *Pseudomonas aeruginosa* are common and increasingly difficult to treat. Pyocins are naturally occurring protein antibiotics produced by *P. aeruginosa* that have potential for human use.

**Objectives:**

To determine if pyocin treatment is effective in a murine model of sepsis with *P. aeruginosa*.

**Methods:**

Recombinant pyocins S5 and AP41 were purified and tested for efficacy in a *Galleria mellonella* infection model and a murine model of *P. aeruginosa* sepsis.

**Results:**

Both pyocins produced no adverse effects when injected alone into mice and showed good *in vitro* antipseudomonal activity. In an invertebrate model of sepsis using *G. mellonella*, both pyocins significantly prolonged survival from 1/10 (10%) survival in controls to 80%–100% survival among groups of 10 pyocin-treated larvae. Following injection into mice, both showed extensive distribution into different organs. When administered 5 h after infection, pyocin S5 significantly increased survival from 33% (2/6) to 83% (5/6) in a murine model of sepsis (difference significant by log-rank test, *P *<* *0.05).

**Conclusions:**

Pyocins S5 and AP41 show *in vivo* biological activity and can improve survival in two models of *P. aeruginosa* infection. They hold promise as novel antimicrobial agents for treatment of MDR infections with this microbe.

## Introduction

*Pseudomonas aeruginosa* is a leading cause of severe hospital-acquired infections, such as ventilator-associated pneumonia, burn wound infections and nosocomial bloodstream infections (BSI). *P. aeruginosa* accounts for 4% of all hospital-acquired BSI cases and has a very high (>30%) mortality rate, as well as high healthcare costs.^1–7^ In addition, because of *P. aeruginosa’*s limited susceptibility to antibiotics and the frequent emergence of resistance during therapy, *P. aeruginosa* infections are difficult to treat.^8^^,^^9^ In common with many bacterial pathogens, there is an increasing prevalence of MDR strains, leading to the classification of *P. aeruginosa* as critical on the WHO list of antibiotic-resistant priority pathogens.^10^

A potential alternative therapeutic strategy to treat MDR is the use of the highly potent and narrow-spectrum bacteriocins as protein antibiotics.^11^ Bacteria produce these very diverse and widespread proteins during intraspecies competition, where they kill only bacteria closely related to the producing strain.^12^^,^^13^ Bacteriocins from *P. aeruginosa* can be classified into different categories, with the most abundant S-type pyocins resembling colicins from *Escherichia coli*.^13^^,^^14^ These multi-domain proteins share a similar structural organization (including a receptor binding domain, a translocation domain and a cytotoxic domain) and are able to efficiently cross the Gram-negative outer membrane through parasitization of nutrient uptake pathways.^15–21^ For pyocin S5, initial binding to the cell surface is mediated by the common polysaccharide antigen and translocation across the outer membrane occurs via the TonB-dependent transporter FptA, which normally functions in ferripyochelin uptake.^15^^,^^16^ The mechanism of pyocin AP41 uptake is poorly understood. The cytotoxic activity of pyocins generally takes the form of a nuclease activity targeting DNA (such as pyocin AP41), tRNA or rRNA, a pore-forming activity targeting the cytoplasmic membrane (pyocin S5) or an enzymatic activity targeting peptidoglycan synthesis.^22^

The potency and specificity of pyocins makes them potential candidates for the treatment of *P. aeruginosa* infection. We previously demonstrated pyocin S5 and AP41 efficacy for the treatment of acute pneumonia in a murine model.^23^ Here we show that pyocin S5 and pyocin AP41 delivered directly to the blood retain their bactericidal activity *in vivo* and display efficacy against a clinical strain of *P. aeruginosa* in a murine sepsis model of infection. Mice provide a good model of human sepsis, displaying many of the same pathological and immunological features following bacterial inoculation.^24^ Additionally, we tested the efficacy of pyocins in treating a high-level antibiotic-resistant infection using a *Galleria mellonella* systemic infection model that has been widely used in studies of bacterial infection and antibiotic efficacy,^25^^,^^26^ highlighting their potential as therapeutics in a world of increasing antimicrobial resistance.

## Materials and methods

### Bacterial strains, plasmids and growth conditions

The plasmid pETPyoAP41, which encodes the genes for pyocin AP41 and the C-terminally His_6_-tagged immunity protein ImAP41 was used for expression of the pyocin AP41–ImAP41 complex as previously described.^23^ pPyoS5 was used for the expression of pyocin S5 (with no affinity tag). This plasmid was constructed by ATUM (Newark, CA, USA) and encodes the pyocin S5 gene, optimized for expression in *E. coli* in the vector pJ404. *P. aeruginosa* strain P7 is a clinical mucoid strain isolated from a CF patient.^23^  *E. coli* strains were cultured at 37°C in LB broth or LB agar supplemented with the appropriate antibiotics. Kanamycin and ampicillin were used at 50 and 100 mg/L, respectively. *P. aeruginosa* strains were cultured under agitation at 37°C in LB broth or LB agar. Methods for the purification of pyocins and the generation of antibodies are available as Supplementary data at *JAC* Online.

### Isolation of a high-level ciprofloxacin-resistant mutant

*P. aeruginosa* strain P7 was grown until OD_600_ = 0.6. Ten times serial dilutions of this culture were then plated on LB agar plates supplemented with 1 mg/L ciprofloxacin (Sigma) and the plates incubated overnight at 37°C. Isolated colonies were then subjected to phenotypic characterization using an overlay spot susceptibility assay and the MIC was determined using standard methods (see below).

### Antibiotic susceptibility assays: MIC

The MIC of antibiotic required to inhibit the visible growth of *P. aeruginosa* isolates was determined using a standard method.^27^ The MIC assay was carried out using serial dilutions in a 96-well plate with 2-fold dilutions.

### G. mellonella larvae infection model

*G. mellonella* larvae were obtained from Livefood UK, kept in darkness at room temperature and were used up to 1 week following arrival. Healthy larvae with no melanization were used for all experiments. *P. aeruginosa* was grown under agitation in LB broth at 37°C to an OD_600_ = 0.6. Cells were then washed twice in sterile PBS and diluted to the desired inoculum in PBS. Inocula were serially diluted and plated on LB agar plates just before administration for cfu counting. Using the approaches outlined in Bell *et al*.,^28^ for a survival proportion of 0.8 in a treated group and 0.2 in the control group, at a power of 95% with a significance level set at 0.01, groups of between 9 and 10 larvae would be required. Groups of 10 larvae were injected with 10 μL of bacterial suspension in the haemocoel via the last right pro-limb. Following challenge, larvae were placed in an incubator at 37°C. Larvae were treated 3 h post-infection by injection of 10 μL of BSA, pyocin or ciprofloxacin in PBS at various concentrations in the haemocoel via the last left pro-limb. Survival was followed for 48 h; larvae were considered dead when unresponsive to touch. For each experiment, groups of uninfected larvae (*n *=* *10) were injected with PBS, BSA, pyocin or antibiotics as a negative control. No deaths were recorded for these control groups. Statistical analysis of Kaplan–Meier survival curves of each group of larvae was performed using the log-rank (Mantel–Cox) test.

### Stability and clearance of pyocins in vivo

LPS-free and sterile filtered pyocin S5 or pyocin AP41 in PBS were injected IV in the tail vein at a concentration of 1 mg/mL in 8–10-week-old female BALB/c mice (ordered from Charles River and left to acclimatize 1 week before use). For each timepoint, two mice were injected per experiment and each experiment was done at least two times. The mice were randomly allocated to each group. Each mouse was marked with a marker on the tail to keep track on the order they were injected, allowing for precise timings. Mice were culled by exposure to CO_2_ at different timepoints following the injection (earliest 10 min, latest 33 h). Blood was obtained via cardiac puncture immediately following CO_2_ asphyxiation and organs were sampled. The blood was left to coagulate for 1 h at room temperature before being centrifuged for 10 min at 3000 rpm to obtain the serum. Treatment of organs was as follows: PBS supplemented with protease inhibitor [1 tablet of cOmplete protease inhibitor (Roche) in 35 mL of PBS] was added to the frozen organs (volume depending on the weight of the organ) before homogenization using a handheld homogenizer (Omni). Homogenized organs were then centrifuged at 4°C for 20 min at 13 000 rpm and supernatant stored with the sera at −80°C until analysis by pyocin susceptibility assay and immunoblots and ELISA. Protocols for pyocin quantification by ELISA and immunoblots and cytokine quantification are available as Supplementary data at *JAC* Online.

### Murine sepsis model

Using the approaches outlined in Bell *et al*.,^28^ for a survival proportion of 0.8 in a treated group and 0.2 in the control group, at a power of 90% with a significance level set at 0.05, groups of between 5 and 7 mice would be required. Female BALB/c mice (7 weeks old; weight range 15–20 g) were obtained from Charles River and randomly allocated to separate cages (M2-type from North Kent Plastics, UK) containing aspen woodchip bedding with a maximum of six mice per cage. Mice were left to acclimatize in the animal unit under a 12:12 h light: dark cycle, with lights on at 7am, for 1 week before use. Groups of six animals were formed randomly prior to the experiment and were infected by IV injection in the tail vein with a lethal dose of exponentially growing *P. aeruginosa* (2 × 10^7^ cfu). Infections always took place at the same time of the day (late mornings) to avoid circadian-rhythm variations. During the experiment, mice had unlimited access to soft baby food and water. Five hours post-infection, mice were treated with LPS-free, sterile filtered BSA or pyocins by IV injection. Clinical health was then monitored for up to 1 week and mice culled by exposure to CO_2_ when reaching the endpoint either when the clinical score set as the threshold (severity score = 7; Table S1, available as Supplementary data at *JAC* Online) was reached or at the predefined endpoint of the experiment. Blood (with or without heparin) and organs were then collected. Statistical analysis of Kaplan–Meier survival curves of each group of mice was performed using the log-rank (Mantel–Cox) test. For cfu counts, blood was serially diluted and plated immediately on LB agar. Organs were homogenized in PBS using a handheld homogenizer (Omni) and plated after serial dilutions. Statistical analysis of cfu counts was performed using the Kruskal–Wallis test followed by Dunn’s multiple comparisons test. Serum was stored at −80°C until analysis.

### Ethics

All animal experiments were performed in accordance with the UK Animals (Scientific Procedures) Act, authorized under a UK Home Office License, and all procedures were approved by the animal project review committee of the University of Glasgow. The project license number assigned by the animal project review committee of the University of Glasgow was P079A1B53.

## Results

### Pyocins S5 and AP41 are bactericidal in different conditions in vitro

**Figure 1.**
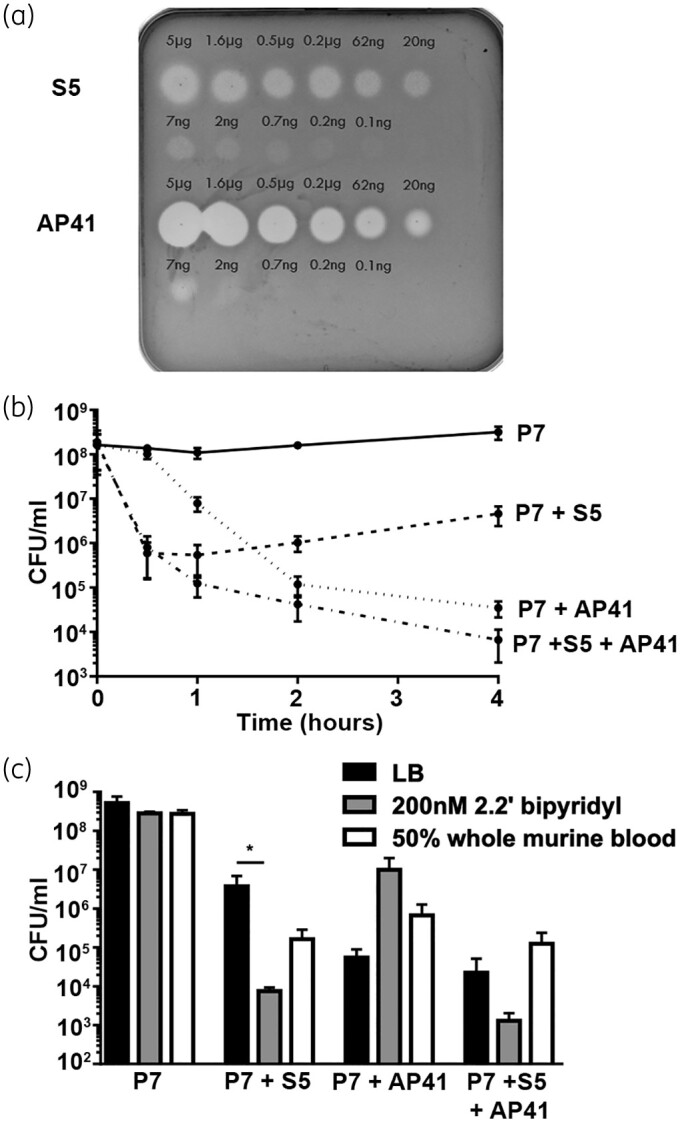
Purified pyocins S5 and AP41 are active *in vitro*. (a) Spot tests to determine the activity of pyocins S5 and AP41 against *P. aeruginosa* strain P7; 5 μL of a range of concentrations of pyocin S5/AP41 was spotted on a growing lawn of *P. aeruginosa*. The presence of clear zones indicates pyocin activity. (b) Bacterial counts over time of *P. aeruginosa* incubated in LB medium supplemented with PBS (control) or 10 mg/L S5, AP41 or S5+AP41. (c) Bacterial counts of *P. aeruginosa* after 4 h of incubation with PBS, S5, AP41 or S5+AP41 (10 mg/L) in different conditions: LB, LB + 200 nM 2.2’ bipyridyl or LB+fresh whole murine blood (50%). Bars represent the average cfu counts in cfu/mL from three experiments. Error bars represent the SD and asterisks indicate significant differences, as assessed by one-way ANOVA (*P <* *0.05).

First, we determined the ability of pyocins S5 and AP41 to kill *P. aeruginosa* strains under different conditions *in vitro*. The pyocin susceptibility spot assay showed that the P7 clinical isolate of *P. aeruginosa* was susceptible to both S5 and AP41, with S5 being more potent [minimum effective dose (MED)  = 0.1 ng] than AP41 (MED = 2 ng) (Figure 1a). In LB liquid culture, both pyocins had bactericidal activity, causing a sharp decrease in cfu counts after 30 min of incubation with S5 and after 1 h of incubation with AP41 (Figure 1b). When a combination of both pyocins was used, a combination of both phenotypes was observed, with a quick but stable decrease in cfu counts over time (Figure 1b). As pore-forming pyocin S5 has been shown to use the ferripyochelin receptor FptA,^16^ the activity of both pyocins was tested in LB supplemented with the iron chelator 2.2’-bipyridyl at a final concentration of 2 mM. As expected, the addition of 2.2’ bipyridyl significantly increased the bactericidal activity of S5 (Figure 1c). However, the addition of the iron chelator had no effect on AP41 activity, suggesting that its unknown receptor is unlikely to be involved in iron uptake. To determine if pyocins retain their bactericidal activity in blood, bacterial counts were followed during incubation with recombinant pyocins in LB supplemented with 50% fresh murine blood (Figure 1c). The presence of blood increased pyocin S5 activity, with a 10-fold decrease in cfu numbers compared with incubation in LB alone. Incubation with AP41 in 50% fresh blood decreased its antimicrobial activity; however, this decrease was not significant (Figure 1c). These results show that both pyocins retain their activity in blood.

### Pyocins S5 and AP41 are able to treat lethal P. aeruginosa infection in G. mellonella larvae

**Figure 2.**
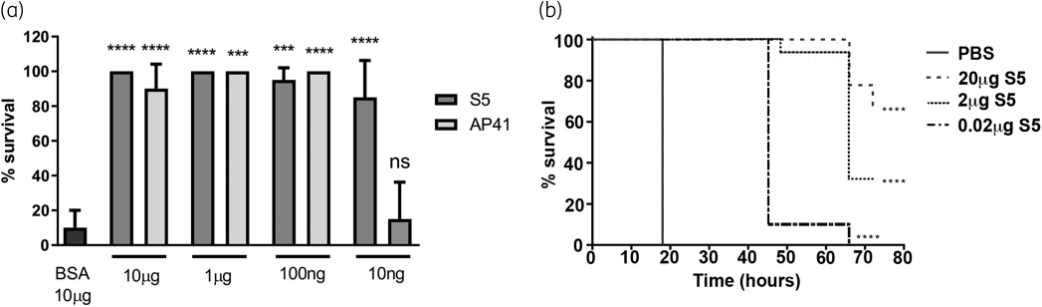
Purified pyocins S5 and AP41 are able to treat *P. aeruginosa*-infected *G. mellonella* larvae. Experiments were performed with 10 larvae per group per experiment. (a) Twenty-four hour survival of larvae infected with *P. aeruginosa* strain P7 and treated 3 h post-infection with BSA, S5 or AP41. Bars represent the mean survival from two experiments (*n *=* *10 larvae per group per experiment). Error bars represent the SD and asterisks indicate significant differences relative to larvae treated with PBS, as assessed by one-way ANOVA (ns, non-significant; ****P <* *0.0001; ***P <* *0.001). (b) Kaplan–Meier survival curves of larvae infected with *P. aeruginosa* strain P7 and treated 3 h post-infection with different doses of S5. Survival curves are representative of two distinct experiments; *n *=* *10 larvae per group per experiment. Asterisks indicate significant differences relative to larvae treated with PBS, as assessed by the log-rank (Mantel–Cox) test (****P <* *0.0001).

To determine if pyocins AP41 and S5 were able to treat a lethal systemic infection *in vivo, G. mellonella* larvae were infected with 5 × 10^3^ cfu of *P. aeruginosa* clinical isolate P7 and treated 3 h following infection with different doses of pyocins. Larvae were monitored and survival assessed 24 h post-infection (Figure 2a). While most untreated larvae were dead by 24 h post-infection, we observed 80% to 100% survival of larvae treated with either S5 (10 ng to 10 μg) or AP41 (100 ng to 10 μg), showing the effectiveness of both pyocins to protect against a lethal *P. aeruginosa* infection. At the lowest treatment dose tested, S5 was able to rescue larvae survival, but there was no increase in survival compared with the untreated larvae when using AP41 (Figure 2a). A separate experiment, extending to 5 days post-infection, showed that, following S5 treatment, survival decreased over time, even at the highest treatment dose (Figure 2b).

### Pyocin S5 is able to treat an antibiotic-resistant P. aeruginosa infection in the Galleria larvae model

**Figure 3.**
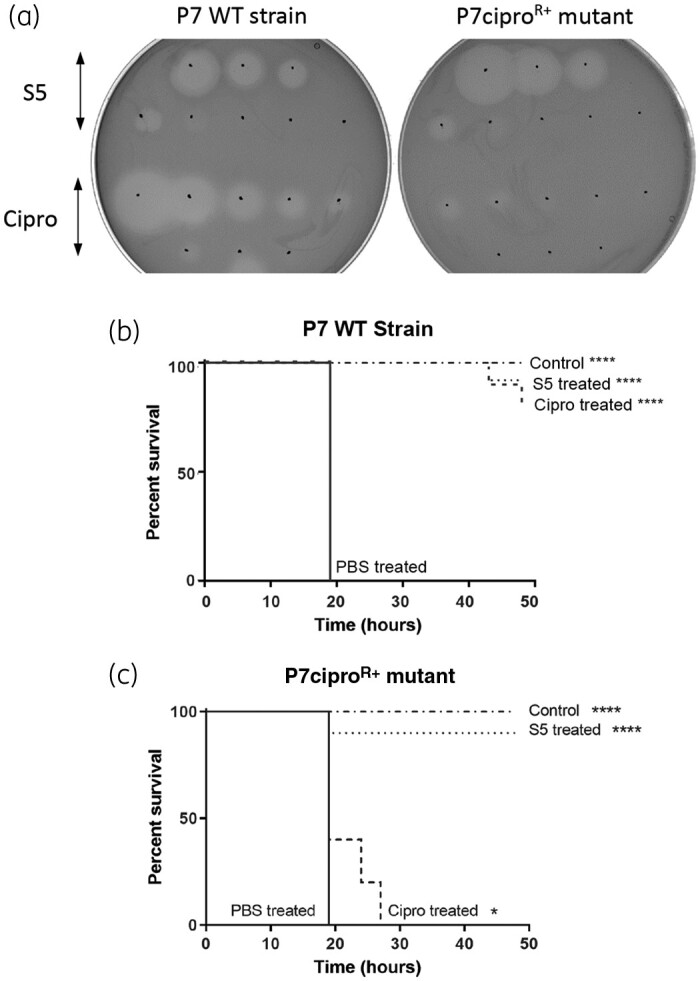
Purified pyocin S5 is active against ciprofloxacin-resistant *P. aeruginosa in vitro* and *in vivo*. (a) Spot tests to determine the activity of ciprofloxacin and pyocin S5 against *P. aeruginosa* strain P7 and a P7-derived ciprofloxacin-resistant mutant. Three microlitres of a range of concentrations of ciprofloxacin (3 μg to 23 ng of ciprofloxacin, 2× dilutions) and pyocin S5 (3 μg to 0.3 pg of pyocin S5, 10× dilutions) was spotted on a growing lawn of *P. aeruginosa*. The presence of clear zones indicates pyocin or antibiotic activity. (b and c) Kaplan–Meier survival curves of larvae infected with the WT *P. aeruginosa* P7 strain (b) or the P7-derived ciprofloxacin-resistant mutant (P7cipro^R+^) (c) (*n *=* *10 per group) treated 3 h post-infection with ciprofloxacin (10 μg) or pyocin S5 (5 μg). Control uninfected larvae (*n *=* *10 per group) were injected with PBS. Groups of uninfected larvae (*n *=* *10 per group) were also injected with ciprofloxacin (10 μg) or pyocin S5 (5 μg) and all survived, but are not represented here. Survival curves are representative of two distinct experiments. Asterisks indicate significant differences relative to larvae treated with PBS, as assessed by the log-rank (Mantel–Cox) test (*P <* *0.05; ****P <* *0.0001).

In order to test the potency of pyocin S5 to treat an infection caused by a high-level antibiotic-resistant strain of *P. aeruginosa*, we generated a high-level ciprofloxacin-resistant mutant (P7cipro^R+^) derived from the clinical isolate P7. The initial P7 isolate is already resistant to ciprofloxacin (MIC = 2 mg/L); the derived high-level resistant mutant had an MIC of 16 mg/L. *In vitro*, this mutant retained its susceptibility to pyocin S5 (Figure 3a). The efficacy of S5 against the P7cipro^R+^ mutant was assessed *in vivo* using the *Galleria* larvae model (Figure 3b and c and Figure S1). As expected, while survival of larvae infected with the WT P7 strain was increased by treatment with ciprofloxacin (10 μg, 80% survival 48 h post-infection), the same treatment was only able to delay the deaths of the larvae infected with the P7cipro^R+^ mutant, with 100% mortality observed 27 h post-infection. S5 was able to rescue the larvae infected with the WT P7 strain or P7cipro^R+^ mutant equally effectively (90% survival 48 h post-infection for both).

### Pyocin dissemination, degradation and activity in mice

**Figure 4.**
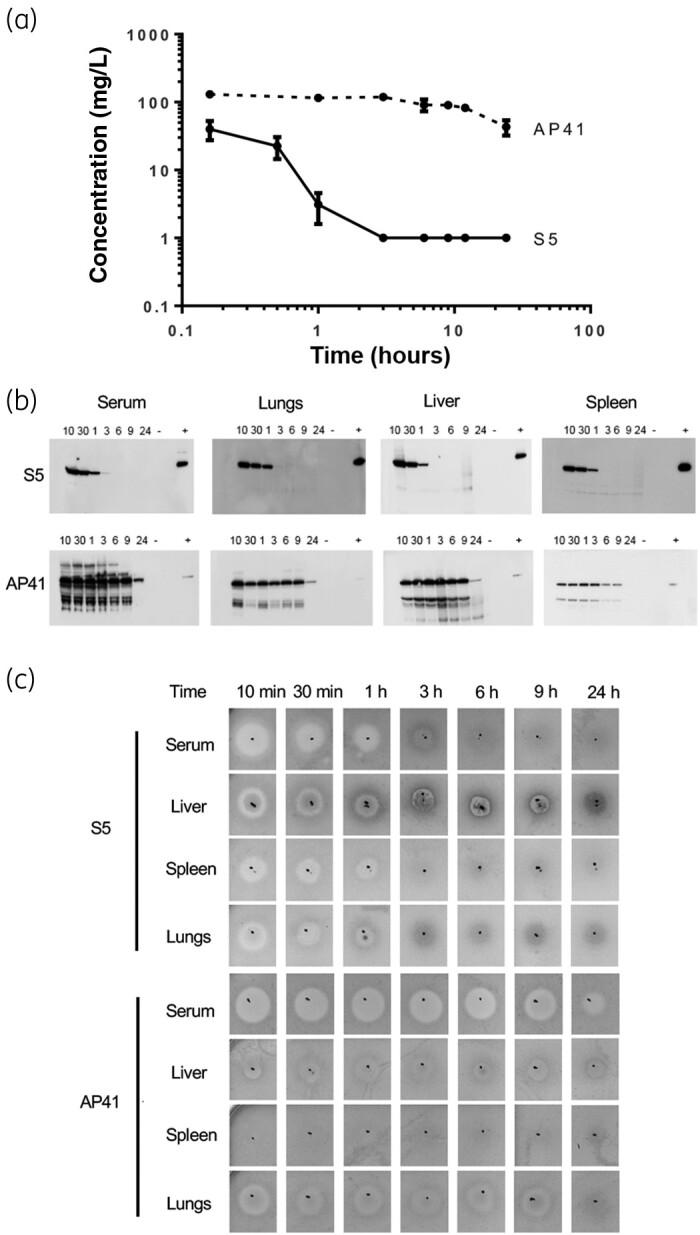
Pyocins S5 and AP41 have different dissemination and clearance profiles in mice. (a) Serum concentration of S5 and AP41 determined by ELISA following IV injection of 200 μg of pyocins in mice. Results represent the mean from three independent experiments, two mice per timepoint and per experiment. Error bars represent the SD. (b) Detection of pyocins S5 and AP41 in serum and organ homogenates at different timepoints (10 min, 30 min, 1 h, 3 h, 6 h, 9 h and 24 h) following injection using S5- or AP41-specific affinity-purified antibodies. Positive control (+) = 1.25 ng of purified S5 or AP41. Negative control (-) = corresponding homogenized organ from a mouse that did not receive injections of S5 or AP41. (c) Detection of pyocin S5 or AP41 activity in serum or homogenized organs at different timepoints following IV injection. Five microlitres of homogenized organ or serum was spotted on a growing lawn of *P. aeruginosa* strain P7. The presence of clear zones indicates pyocin activity.

To begin exploring the utility of pyocins in treating *P. aeruginosa* blood infections, we first investigated their distribution, clearance and stability *in vivo*. High doses of AP41 or S5 (200 μg) were injected IV into mice and the concentrations of AP41 and S5 in serum over time were determined by ELISA using polyclonal specific AP41 or S5 antibodies, respectively (Figure 4a). The serum concentration of S5 decreased sharply almost immediately following injection, being detectable in the serum only up to 1 h post-injection. By contrast, the serum concentration of AP41 only slightly decreased over time. Dissemination of S5 and AP41 from the blood to different organs was then determined by western blot (Figure 4b). Pyocin S5 was detected up to 1 h following injection in serum, confirming the ELISA results, as well as in the spleen, lungs and liver, showing good distribution of the pyocin. Pyocin AP41 was detected up to 24 h post-injection in serum, spleen, lungs and liver, showing good distribution and stability. Finally, we tested the killing activity against *P. aeruginosa* of both pyocins from the same samples (Figure 4c). As expected, zones of inhibition were detected up to 1 h post-injection in serum, lungs and spleen of S5-treated mice and up to 24 h in serum and lungs of AP41-treated mice. The antibacterial activity demonstrated using the susceptibility assay correlates well with results from both western blot and ELISA techniques, other than a lack of bactericidal activity in the spleens of AP41-treated mice. To determine if injection of pyocins on their own induces a pro- or anti-inflammatory response in non-infected mice when injected IV, we quantified levels of pro-inflammatory (IL-1β and TNF-ɑ) and anti-inflammatory (IL-10) cytokines in serum samples from S5- and AP41-treated mice and compared these with the levels observed in non-injected mice. Neither pyocin S5 nor pyocin AP41 induced production of these cytokines, with levels in treated animals remaining very similar to those of control mice (Figure S2).

### Pyocins S5 and AP41 improve survival of septic mice

**Figure 5.**
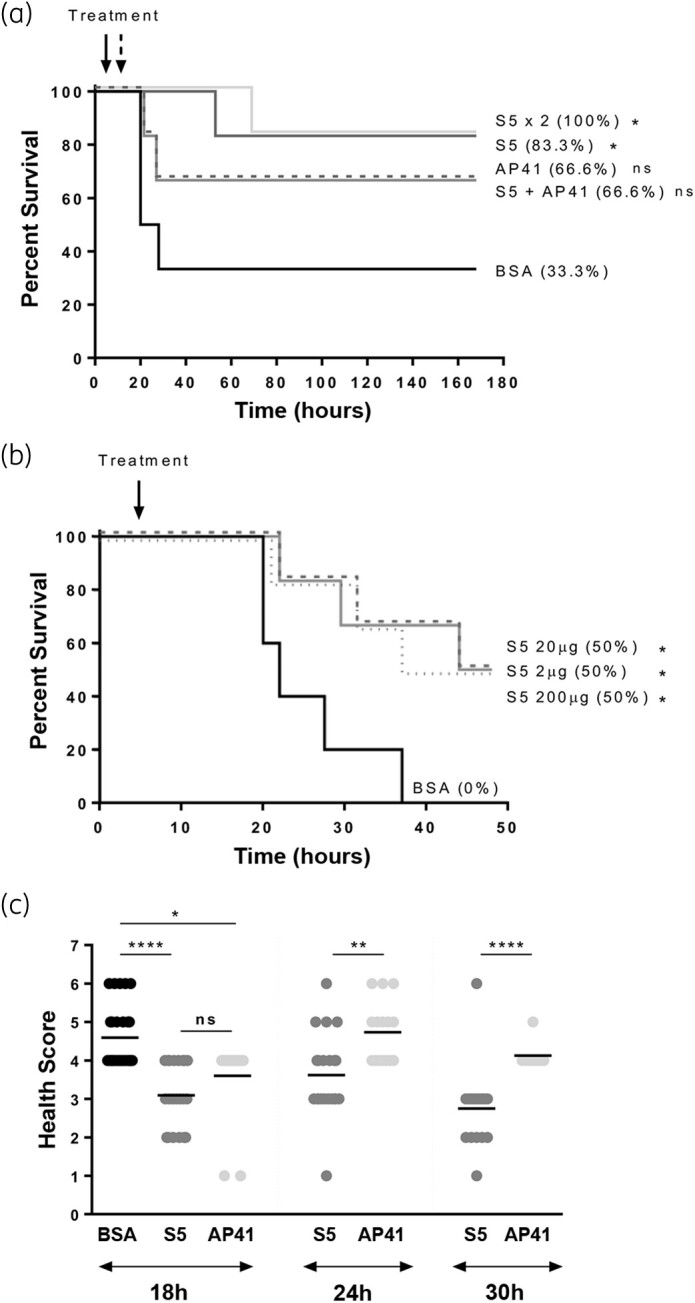
Pyocins S5 and AP41 improve survival of septic mice. For each experiment the number of mice per individual treated group was six. (a) Kaplan–Meier survival curves of mice (*n *=* *6 per group) infected IV with 5.6 × 10^7^ cfu of *P. aeruginosa* strain P7 and treated with IV injection 5 h post-infection with a single injection of 2 μg of BSA, S5 or AP41, with an injection of a cocktail of S5+AP41 (2 μg) or with two injections of 2 μg of S5 (5 and 11 h post-infection). (b) Kaplan–Meier survival curves of mice infected IV with 4 × 10^7^ cfu of *P. aeruginosa* strain P7 and treated with IV injection 5 h post-infection with 200 μg (dotted line), 20 μg (dashed line) or 2 μg (solid line) of S5 or 200 μg of BSA. Survival curves are representative of at least two independent experiments, *n *=* *6 per group. Asterisks indicate significant differences relative to mice treated with BSA, as assessed by the log-rank (Mantel–Cox) test (ns, non-significant; *P <* *0.05). (c) Clinical scores of infected mice at 18, 24 and 30 h post-infection. Data were collected from four different experiments, *n *=* *6 per group. Asterisks indicate significant differences, as assessed by one-way ANOVA (ns, non-significant; *P <* *0.05; **P <* *0.01; ****P <* *0.0001).

To determine if pyocins S5 and AP41 are able to improve survival rates of septic mice when administered post-infection, 7-week-old mice were infected by IV injection of a lethal dose of strain P7 and treated with IV injection of 2 μg of pyocin S5 or AP41 at 5 h post-infection. Representative experiments are shown in Figure 5. Pyocins S5 and AP41 were able to increase survival in this model from 33% to 83% and 67%, respectively (Figure 5a); this is significant for S5, but not AP41 (log-rank test, *P *<* *0.05). A second injection of 2 μg of S5 did not improve the survival rate; in addition, the use of a cocktail of S5 and AP41 did not improve the survival rate. To determine if increasing the concentration would result in increased survival of infected mice, different doses of pyocin S5 were used (200, 20 and 2 μg) and the mice monitored for 72 h. Treatment with 2, 20 or 200 μg of pyocin S5 had a very similar effect on survival (Figure 5b).

Although there was an increase in survival for the mice treated with pyocin S5 or AP41, they still displayed signs of infection. Following infection, mice were monitored regularly and a health score given to each of them using a scoring system for disease severity (Table S1 and Figure 5c). The onset of the disease for sham-treated mice occurred between 18 and 24 h post-infection. At 18 h post-infection, mice were given an average score of 4.6, 3.0 and 3.6 for sham-treated, S5-treated and AP41-treated mice, respectively; a significant reduction at that timepoint for pyocin-treated mice. As most sham-treated mice did not survive beyond the 18 h post-infection timepoint, no clinical scores were included for that group after this timepoint. Comparison of the health scores of S5- and AP41-treated mice 24 and 30 h post-infection shows significantly lower health scores and a more rapid recovery of S5-treated mice. The mice were monitored for up to 7 days following infection and, once completely recovered, no relapse was observed and there were no delayed murine deaths. Measurement of the concentrations of serum pro- and anti-inflammatory cytokines (IL-1β, IFN-γ, TNF-ɑ and IL-10) quantified for each mouse before infection, after infection and after pyocin injection showed there was no significant difference in cytokine levels following treatment of infected mice with BSA, pyocin S5 or pyocin AP41 (Figure S3).

**Figure 6.**
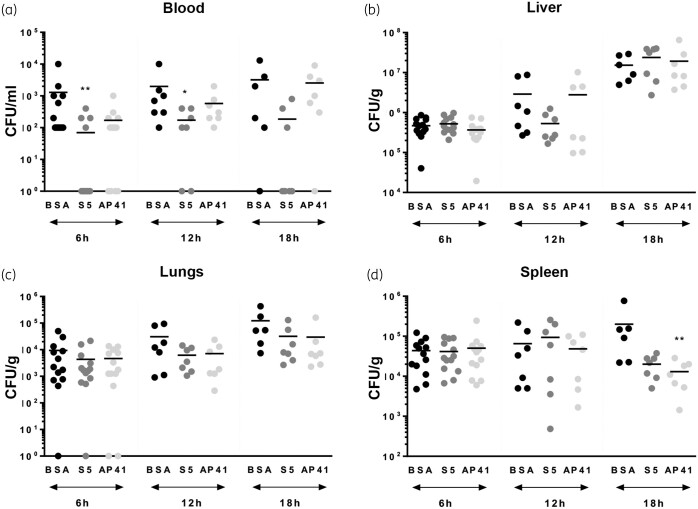
Bacterial counts in pyocin-treated mice. Bacterial counts 6, 12 and 18 h post-infection in the blood (a), liver (b), lungs (c) and spleen (d) of infected mice treated with 2 μg of BSA, 2 μg of S5 or 2 μg of AP41. Asterisks indicate significant differences relative to sham-treated mice, as assessed by the Kruskal–Wallis test followed by Dunn’s multiple comparisons test (*P <* *0.05; **P <* *0.01). When not indicated, the differences were not statistically different.

To determine if pyocins S5 and AP41 are able to reduce bacterial load in mice following infection, groups of six mice were culled 6, 12 and 19 h post-infection and bacterial counts in blood, liver, lungs and spleen from treated mice were compared with those of sham-treated mice (Figure 6). In these experiments, S5 significantly reduced bacterial load in the blood at 6 and 12 h post-infection. Differences between groups were tested by the Kruskal–Wallis test and were significant (*P *<* *0.05) and individual timepoint differences were assessed by Dunn’s multiple comparison test and were significant as shown (*P *<* *0.05). However, the reduction in bacterial numbers in the blood of AP41-treated mice was not significant (Figure 6a). As both bacteria and pyocins will disseminate from the blood to organs, we also determined bacterial load in the organs of untreated and treated mice. There was no significant reduction in bacterial numbers in the liver and lungs of pyocin-treated mice (Figure 6b and c). In the spleen, we observed a significant decrease in cfu counts 19 h post-infection for AP41-treated mice (Figure 6d). No bacteria were found in the blood of mice that recovered from infection after being treated with 2 μg of S5 (*n *=* *6, *t *=* *72 h post-infection; *n *=* *5, *t *=* *168 h post-infection) or with 2 μg of AP41 (*n *=* *6, *t *=* *72 h post-infection; *n *=* *5, *t *=* *168 h post-infection). To determine if pyocin tolerance was acquired following IV treatment of septic mice, cfu recovered from mice at different timepoints following infection were tested for pyocin susceptibility (*n = *119; Table S2). No difference in susceptibility to pyocin S5 or AP41 was observed in colonies recovered from mice compared with that of the original P7 strain.

## Discussion

In this study, single injections of two *P. aeruginosa*-specific pyocins are shown to improve the survival of septic mice. When injected directly in the blood, pyocins do not provoke an immune reaction, remain stable and retain their activity for a minimum of 1 h for S5 and 24 h for AP41. We demonstrate that both pyocins are able to disseminate into the liver, the spleen and the heart, where they are active. Surprisingly, while AP41 is found to remain stable and active in the blood and organs for a longer period *in vivo*, and found to be more efficient for colony-count reduction *in vitro*, it is actually less efficient as treatment in a murine model of sepsis. Additionally, we saw no significant reduction in cfu counts in the blood of infected mice following treatment with AP41. Interestingly, the difference in efficacy of treatment with S5 and AP41 in infected mice was also observed in infected larvae, further validating the use of *G. mellonella* larvae as a bridge between *in vitro* assay and murine infection models. These observations could be due to a mechanistic difference between AP41 and S5 killing or an interaction of AP41 with host proteins, decreasing its activity *in vivo* against *P. aeruginosa*. Further studies would be necessary to test these different hypotheses.

A single injection of pyocin is not sufficient to produce a 100% survival rate, although the bacteria recovered from animals following infection and treatment were not resistant or tolerant to the pyocins. It is possible that giving a continuous IV infusion of pyocin will increase the survival rate, although a second injection of pyocin S5 at 12 h post-infection did not improve survival compared with a single injection.

Taken together, our results show that pyocins can act as effective agents in the animal models of sepsis used in these studies and further investigations of their potential for use as targeted therapeutics in *P. aeruginosa* infections are warranted.

## Funding

The work was funded by the Wellcome Trust (grant number 201505/Z/16/Z), the MRC (grant number MC_PC_18048) and Tenovus Scotland (grant number S19-04). The funders did not play any role in the experimental design or presentation of the results reported here. 

## Transparency declarations

The University of Glasgow has filed a patent on the use of pyocins to treat *P. aeruginosa* lung infection with D.W. as an inventor. There are no other conflicts of interest for any of the authors.

## Supplementary data

Supplementary Methods, Tables S1 and S2 and Figures S1 to S3 are available as Supplementary data at *JAC* Online.

## Supplementary Material

dkab199_Supplementary_Data

## References

[1] Kang C-I, Kim S-H, Park WB et al Bloodstream infections caused by antibiotic-resistant gram-negative bacilli: risk factors for mortality and impact of inappropriate initial antimicrobial therapy on outcome. Antimicrob Agents Chemother 2005; 49: 760–6. 15673761 10.1128/AAC.49.2.760-766.2005 PMC547233

[2] Vidal F, Mensa J, Almela M et al Epidemiology and outcome of *Pseudomonas aeruginosa* bacteremia, with special emphasis on the influence of antibiotic treatment. Analysis of 189 episodes. Arch Intern Med 1996; 156: 2121–6. 8862105

[3] Nathwani D, Raman G, Sulham K et al Clinical and economic consequences of hospital-acquired resistant and multidrug-resistant *Pseudomonas aeruginosa* infections: a systematic review and meta-analysis. Antimicrob Resist Infect Control 2014; 3: 32. 25371812 10.1186/2047-2994-3-32 PMC4219028

[4] Johnson LE, D’Agata EMC, Paterson DL et al *Pseudomonas aeruginosa* bacteremia over a 10-year period: multidrug resistance and outcomes in transplant recipients. Transpl Infect Dis 2009; 11: 227–34. 19302282 10.1111/j.1399-3062.2009.00380.x

[5] Suárez C, Peña C, Gavaldà L et al Influence of carbapenem resistance on mortality and the dynamics of mortality in *Pseudomonas aeruginosa* bloodstream infection. Int J Infect Dis 2010; 14 Suppl 3: e73–8. 10.1016/j.ijid.2009.11.019 20223693

[6] Morata L, Cobos-Trigueros N, Martínez JA et al Influence of multidrug resistance and appropriate empirical therapy on the 30-day mortality rate of *Pseudomonas aeruginosa* bacteremia. Antimicrob Agents Chemother 2012; 56: 4833–7. 22751533 10.1128/AAC.00750-12 PMC3421866

[7] Hattemer A, Hauser A, Diaz M et al Bacterial and clinical characteristics of health care- and community-acquired bloodstream infections due to *Pseudomonas aeruginosa*. Antimicrob Agents Chemother 2013; 57: 3969–75. 23733476 10.1128/AAC.02467-12 PMC3719744

[8] Livermore DM. Multiple mechanisms of antimicrobial resistance in *Pseudomonas aeruginosa*: our worst nightmare? Clin Infect Dis 2002; 34: 634–40. 11823954 10.1086/338782

[9] Carmeli Y, Troillet N, Eliopoulos GM et al Emergence of antibiotic-resistant *Pseudomonas aeruginosa*: comparison of risks associated with different antipseudomonal agents. Antimicrob Agents Chemother 1999; 43: 1379–82. 10348756 10.1128/aac.43.6.1379 PMC89282

[10] WHO. *Prioritization of Pathogens to Guide Discovery, Research and Development of New Antibiotics for Drug Resistant Bacterial Infections, Including Tuberculosis*. 2017.

[11] Behrens HM, Six A, Walker D et al The therapeutic potential of bacteriocins as protein antibiotics. Emerg Top Life Sci 2017; 1: 65–74. 33525816 10.1042/ETLS20160016 PMC7243282

[12] Hammami R, Fernandez B, Lacroix C et al Anti-infective properties of bacteriocins: an update. Cell Mol Life Sci 2013; 70: 2947–67. 23109101 10.1007/s00018-012-1202-3 PMC11113238

[13] Sharp C, Bray J, Housden NG et al Diversity and distribution of nuclease bacteriocins in bacterial genomes revealed using Hidden Markov Models. PLOS Comput Biol 2017; 13: e1005652. 28715501 10.1371/journal.pcbi.1005652 PMC5536347

[14] Ghequire MGK, De Mot R. Ribosomally encoded antibacterial proteins and peptides from Pseudomonas. FEMS Microbiol Rev 2014; 38: 523–68. 24923764 10.1111/1574-6976.12079

[15] Behrens HM, Lowe ED, Gault J et al Pyocin S5 import into *Pseudomonas aeruginosa* reveals a generic mode of bacteriocin transport. mBio 2020; 11: e03230-19. 32156826 10.1128/mBio.03230-19 PMC7064778

[16] Elfarash A, Dingemans J, Ye L et al Pore-forming pyocin S5 utilizes the FptA ferripyochelin receptor to kill *Pseudomonas aeruginosa*. Microbiology (Reading) 2014; 160: 261–9. 24217175 10.1099/mic.0.070672-0

[17] Elfarash A, Wei Q, Cornelis P. The soluble pyocins S2 and S4 from *Pseudomonas aeruginosa* bind to the same FpvAI receptor. Microbiologyopen 2012; 1: 268–75. 23170226 10.1002/mbo3.27 PMC3496971

[18] Denayer S, Matthijs S, Cornelis P. Pyocin S2 (Sa) kills *Pseudomonas aeruginosa* strains via the FpvA type I ferripyoverdine receptor. J Bacteriol 2007; 189: 7663–8. 17720787 10.1128/JB.00992-07 PMC2168733

[19] McCaughey LC, Josts I, Grinter R et al Discovery, characterization and *in vivo* activity of pyocin SD2, a protein antibiotic from *Pseudomonas aeruginosa*. Biochem J 2016; 473: 2345–58. 27252387 10.1042/BCJ20160470 PMC4964976

[20] Baysse C, Meyer JM, Plesiat P et al Uptake of pyocin S3 occurs through the outer membrane ferripyoverdine type II receptor of *Pseudomonas aeruginosa*. J Bacteriol 1999; 181: 3849–51. 10368165 10.1128/jb.181.12.3849-3851.1999 PMC93868

[21] Atanaskovic I, Mosbahi K, Sharp C et al Targeted killing of *Pseudomonas aeruginosa* by pyocin G occurs via the hemin transporter Hur. J Mol Biol 2020; 432: 3869–80. 32339530 10.1016/j.jmb.2020.04.020 PMC7322526

[22] Michel-Briand Y, Baysse C. The pyocins of *Pseudomonas aeruginosa*. Biochimie 2002; 84: 499–510. 12423794 10.1016/s0300-9084(02)01422-0

[23] McCaughey LC, Ritchie ND, Douce GR et al Efficacy of species-specific protein antibiotics in a murine model of acute *Pseudomonas aeruginosa* lung infection. Sci Rep 2016; 6: 30201. 27444885 10.1038/srep30201 PMC4957109

[24] Tracey KJ, Fong Y, Hesse DG et al Anti-cachectin/TNF monoclonal antibodies prevent septic shock during lethal bacteraemia. Nature 1987; 330: 662–4. 3317066 10.1038/330662a0

[25] Tsai CJ-Y, Loh JMS, Proft T. *Galleria mellonella* infection models for the study of bacterial diseases and for antimicrobial drug testing. Virulence 2016; 7: 214–29. 26730990 10.1080/21505594.2015.1135289 PMC4871635

[26] Six A, Kranjangwong S, Crumlish M et al *Galleria mellonella* as an infection model for the multi-host pathogen *Streptococcus agalactiae* reflects hypervirulence of ST283. bioRxiv 2018; doi:10.1101/407171. 10.1080/21505594.2019.1631660 PMC6592362 31230520

[27] Chousterman BG, Swirski FK, Weber GF. Cytokine storm and sepsis disease pathogenesis. Semin Immunopathol 2017; 39: 517–28. 28555385 10.1007/s00281-017-0639-8

[28] Bell ML, Teixeira-Pinto A, McKenzie JE et al A myriad of methods: calculated sample size for two proportions was dependent on the choice of sample size formula and software. J Clin Epidemiol 2014; 67: 601–5. 24439070 10.1016/j.jclinepi.2013.10.008

