# Supplementary material for: Pyocin efficacy in a murine model of Pseudomonas aeruginosa sepsis
Source: J Antimicrob Chemother. 2021 Jun 18;76(9):2317–24. doi: 10.1093/jac/dkab199 (PMC8361349; doi:10.1093/jac/dkab199)
Supplement: dkab199_Supplementary_Data [file dkab199_supplementary_data.docx]

**Supplementary data**


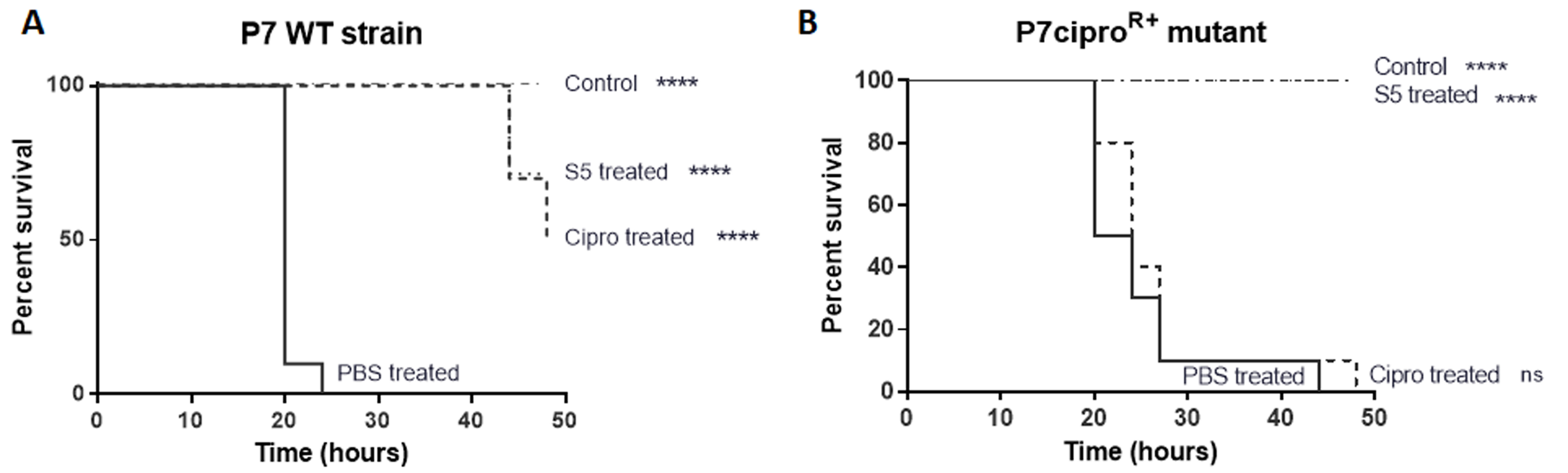


**Figure S1. Purified pyocin S5 is efficient against high-level ciprofloxacin-resistant *Pseudomonas aeruginosa* *in vivo***. Kaplan-Meier survival curves of larvae infected with the WT *P. aeruginosa* P7 strain **(A)** or the P7-derived ciprofloxacin-resistant mutant (P7ciproR+) **(B)** (n=10 per group) treated 3 h post-infection with ciprofloxacin (10 µg) or pyocin S5 (5 µg). Control uninfected larvae (n=10 per group) were injected with PBS. Groups of uninfected larvae (n=10 per group) were also injected with ciprofloxacin (10 µg) or pyocin S5 (5 µg) and all survived but are not represented here. Survival curves are representative of two distinct experiments. Asterisks indicate significant differences relative to larvae treated with PBS, as assessed by the log-rank (Mantel-Cox) test (ns: non-significant; ****P<0.0001).


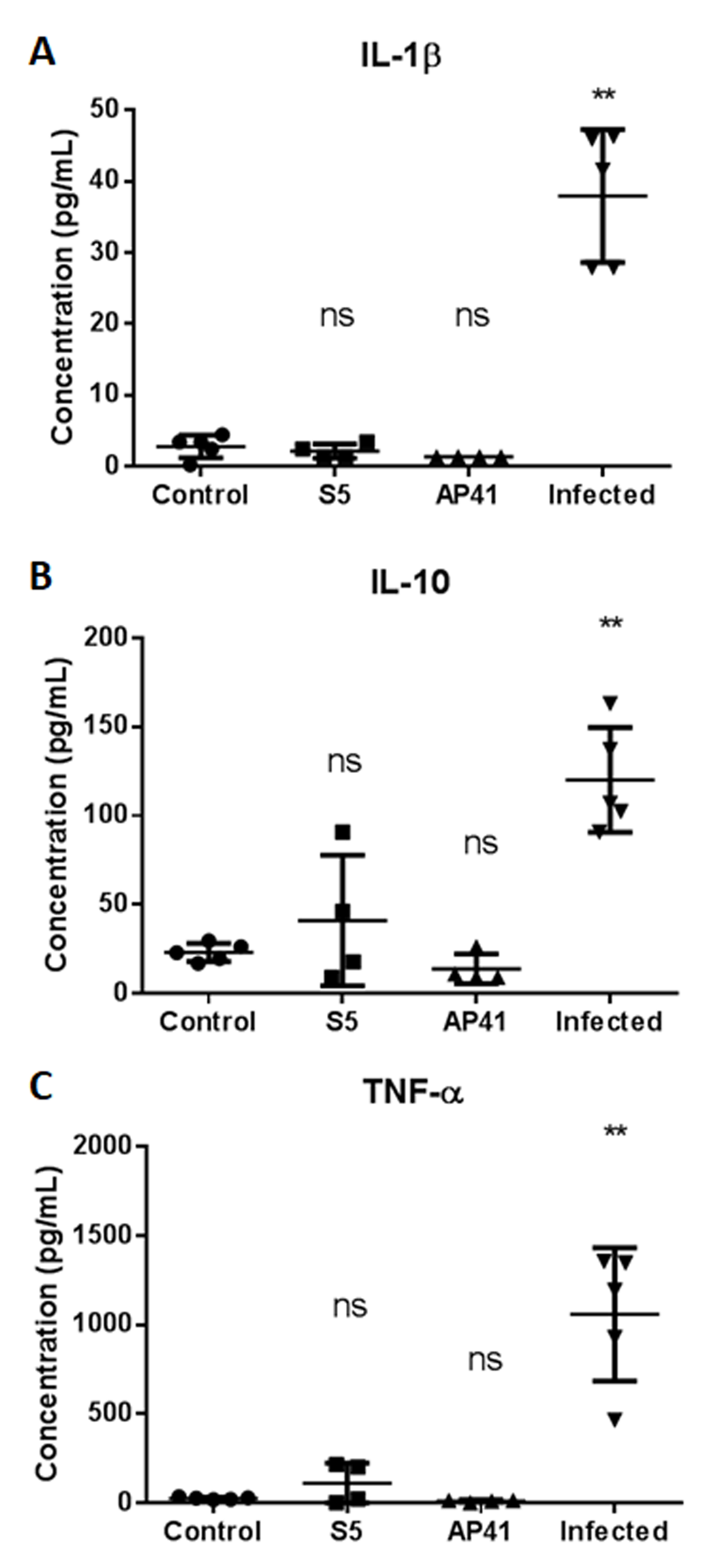


**Figure S2. Effect of Pyocins S5 and AP41 on cytokine production.** Serum levels of cytokines were quantified from mice having received an IV injection of S5 or AP41 (200 µg, 1 h post-injection), from un-injected healthy mice (negative control) and from mice infected by IV with *P. aeruginosa* P7 strain (2 h post-infection). Data points represent levels of IL-1β (A), IL-10 (B) and TNF-ɑ (C) obtained for each mouse; the bar shows the mean value, and the error bars the SD. Asterisks indicate significant differences as assessed by one-way ANOVA (ns: non-significant; **: P<0.01).


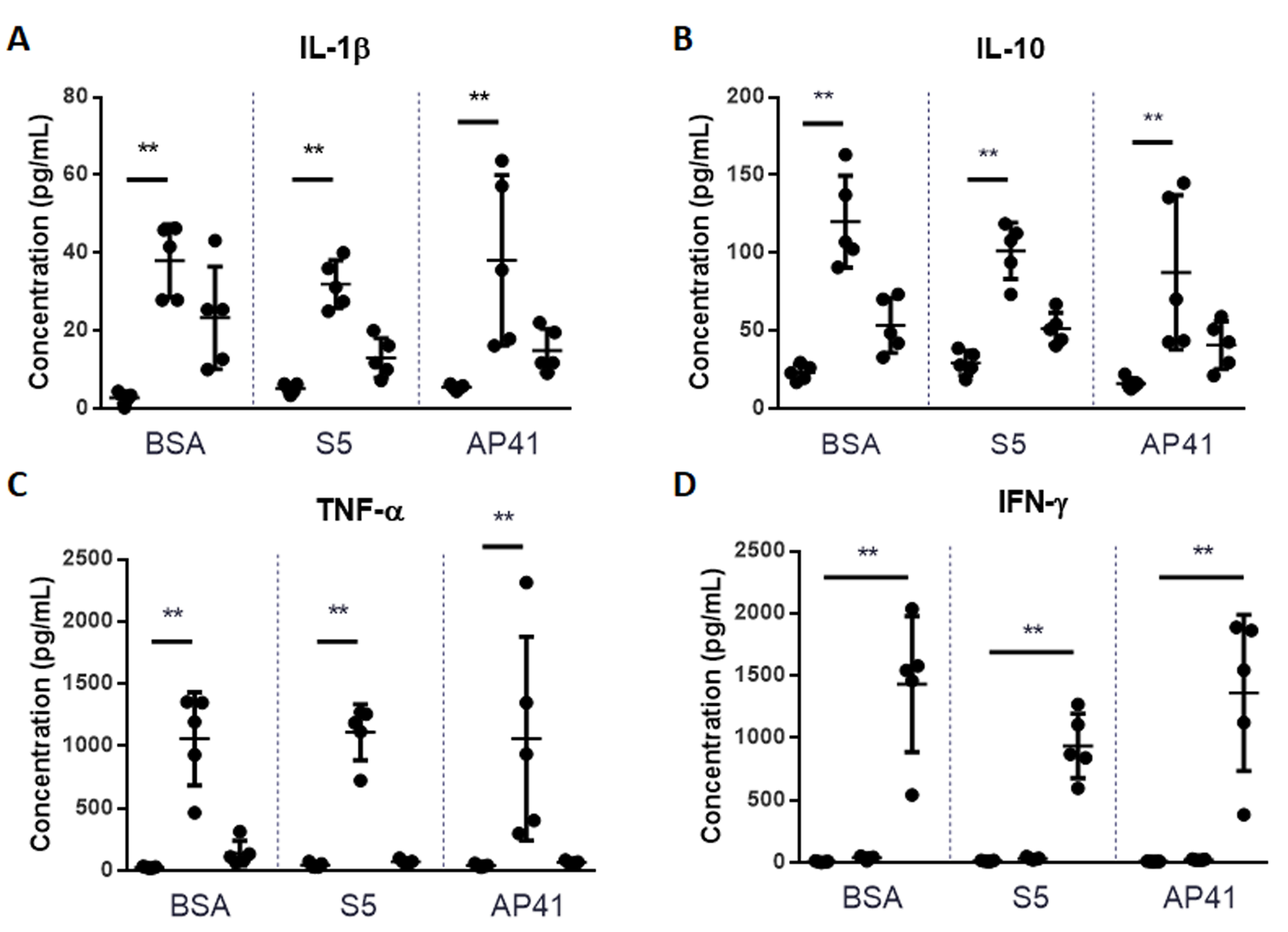


**Figure S3. Treatment of septic mice with pyocin S5 or AP41 does not alter cytokine production.** Serum concentration of IL-1β **(A)**, IL-10 **(B)**, TNF-ɑ **(C)** and IFN-γ **(D)** in mice one-hour post-treatment. Each symbol represents the mean value obtained per mouse. The bar shows the mean value, and the error bars the SD Asterisks indicate significant differences, as assessed by the Kruskal-Wallis test followed by Dunn’s multiple comparisons test (** P<0.01). When not indicated, the differences were not statistically different.

| **Table S1.** Murine health scoring | |  |
| --- | --- | --- |
|  |  |  |
| **Condition** | **Description** | **Overall severity score** |
| Normal | Normal stance and movement | 0 |
| Hunched | Slightly hunched stance | 1 |
|  | Pronounced hunched stance | 2 |
| Starey coat | Mild piloerection of coat, mainly around back of neck | 3 |
|  | Marked piloerection over whole body | 4 |
| Lethargic | Slightly slower movement than usual | 5 |
|  | Obviously slower movement | 6 |
| Moribund | Unwillingness to move when encouraged to do so | 7 |

| **Table S2.** Origin of the CFU recovered from mice tested for pyocin sensitivity | | |
| --- | --- | --- |
|  |  |  |
| **Strain ID** | **Treatment** | **Origin** |
| AS1 | AP41 200µg | Blood |
| AS2 | AP41 200µg | Blood |
| AS3 | AP41 200µg | Blood |
| AS4 | AP41 200µg | Blood |
| AS5 | AP41 200µg | Blood |
| AS6 | AP41 200µg | Blood |
| AS7 | AP41 200µg | Blood |
| AS8 | AP41 200µg | Blood |
| AS9 | AP41 20µg | Blood |
| AS10 | AP41 20µg | Blood |
| AS11 | AP41 20µg | Blood |
| AS12 | AP41 20µg | Blood |
| AS13 | AP41 20µg | Blood |
| AS14 | AP41 20µg | Blood |
| AS15 | AP41 20µg | Blood |
| AS16 | AP41 20µg | Blood |
| AS17 | AP41 2µg | Blood |
| AS18 | AP41 2µg | Blood |
| AS19 | AP41 2µg | Blood |
| AS20 | AP41 2µg | Blood |
| AS21 | AP41 2µg | Blood |
| AS22 | AP41 2µg | Blood |
| AS23 | AP41 2µg | Blood |
| AS24 | AP41 2µg | Blood |
| AS25 | BSA 200µg | Blood |
| AS26 | BSA 200µg | Blood |
| AS27 | BSA 200µg | Blood |
| AS28 | BSA 200µg | Blood |
| AS29 | BSA 200µg | Blood |
| AS30 | BSA 200µg | Blood |
| AS31 | BSA 200µg | Blood |
| AS32 | BSA 200µg | Blood |
| AS33 | S5 2µg | Liver |
| AS34 | S5 2µg | Liver |
| AS35 | S5 2µg | Lungs |
| AS36 | S5 2µg | Lungs |
| AS37 | S5 2µg | Lungs |
| AS38 | S5 2µg | Spleen |
| AS39 | S5 2µg | Spleen |
| AS40 | S5 2µg | Blood |
| AS41 | S5 2µg | Blood |
| AS42 | S5 2µg | Blood |
| AS43 | AP41 2µg | Liver |
| AS44 | AP41 2µg | Liver |
| AS45 | AP41 2µg | Liver |
| AS46 | AP41 2µg | Lungs |
| AS47 | AP41 2µg | Lungs |
| AS48 | AP41 2µg | Lungs |
| AS49 | AP41 2µg | Lungs |
| AS50 | AP41 2µg | Lungs |
| AS51 | AP41 2µg | Spleen |
| AS52 | AP41 2µg | Blood |
| AS53 | BSA 2µg | Spleen |
| AS54 | BSA 2µg | Blood |
| AS55 | BSA 2µg | Liver |
| AS56 | S5 2µg | Liver |
| AS57 | S5 2µg | Liver |
| AS58 | AP41 2µg | Liver |
| AS59 | AP41 2µg | Liver |
| AS60 | S5+AP41 2µg | Liver |
| AS61 | S5+AP41 2µg | Liver |
| AS62 | S5+AP41 2µg | Liver |
| AS63 | S5+AP41 2µg | Liver |
| AS64 | Gentamicin 2µg | Liver |
| AS65 | Gentamicin 2µg | Liver |
| AS66 | BSA 2µg | Liver |
| AS67 | BSA 2µg | Blood |
| AS68 | BSA 2µg | Lungs |
| AS69 | BSA 2µg | Spleen |
| AS70 | S5 2µg | Liver |
| AS71 | S5 2µg | Blood |
| AS72 | S5 2µg | Lungs |
| AS73 | S5 2µg | Spleen |
| AS74 | AP41 2µg | Liver |
| AS75 | AP41 2µg | Blood |
| AS76 | AP41 2µg | Lungs |
| AS77 | AP41 2µg | Spleen |
| AS78 | S5+AP41 2µg | Liver |
| AS79 | S5+AP41 2µg | Blood |
| AS80 | S5+AP41 2µg | Lungs |
| AS81 | S5+AP41 2µg | Spleen |
| AS82 | S5 2µg | Spleen |
| AS83 | S5 2µg | Spleen |
| AS84 | S5 2µg | Spleen |
| AS85 | S5 2µg | Spleen |
| AS86 | S5 2µg | Spleen |
| AS87 | S5 2µg | Lungs |
| AS88 | S5 2µg | Lungs |
| AS90 | S5 2µg | Lungs |
| AS92 | AP41 2µg | Spleen |
| AS93 | AP41 2µg | Spleen |
| AS94 | AP41 2µg | Spleen |
| AS95 | AP41 2µg | Spleen |
| AS96 | AP41 2µg | Spleen |
| AS97 | AP41 2µg | Lungs |
| AS98 | AP41 2µg | Lungs |
| AS99 | AP41 2µg | Lungs |
| AS100 | AP41 2µg | Lungs |
| AS101 | AP41 2µg | Lungs |
| AS102 | S5 2µg | Blood |
| AS103 | S5 2µg | Lungs |
| AS104 | S5 2µg | Lungs |
| AS105 | S5 2µg | Lungs |
| AS106 | S5 2µg | Lungs |
| AS107 | S5 2µg | Spleen |
| AS108 | S5 2µg | Spleen |
| AS109 | S5 2µg | Liver |
| AS110 | S5 2µg | Liver |
| AS111 | S5 2µg | Liver |
| AS112 | AP41 2µg | Blood |
| AS113 | AP41 2µg | Lungs |
| AS114 | AP41 2µg | Lungs |
| AS115 | AP41 2µg | Lungs |
| AS116 | AP41 2µg | Lungs |
| AS117 | AP41 2µg | Spleen |
| AS118 | AP41 2µg | Spleen |
| AS119 | AP41 2µg | Liver |
| AS120 | AP41 2µg | Liver |
| AS121 | AP41 2µg | Liver |

**Supplementary Methods**

***Purification of pyocins and generation of antibodies***

Pyocin S5 was overexpressed from *E. coli* BL21 (DE3) carrying the plasmid pPyoS5 with initial purification using a cation exchange column (CM16/10 FF, GE Healthcare; buffers: sodium phosphate 50 mM pH 6; sodium phosphate 50 mM pH 6 + NaCl 500 mM). Remaining contaminants were removed using an anion exchange column (DEAE 16/10 FF, GE Healthcare; buffers: Tris 20 mM pH 8; Tris 20 mM pH 8 + 500 mM NaCl). The pyocin AP41-ImAP41 complex was purified by nickel affinity chromatography and gel filtration as described previously^24^. For both pyocins, contaminating endotoxins were removed, prior to first storage at -80°C, using endotoxin removal spin columns (Thermo Scientific #88274). LPS concentration was then verified using the Pierce LAL chromogenic endotoxin quantitation kit (Thermo Scientific 88282). Purified S5 was considered LPS-free when the levels detected were lower than that of the lowest standard of the kit at 0.1 EU/mL. Methods for *in vitro* pyocin activity assays are provided in the supplementary material.

***Immunoblots***

Proteins were separated by SDS-PAGE and then transferred on PVDF membranes using the Trans-Blot Turbo blotting system (Biorad). Pyocins S5 and AP41 were detected using rabbit specific polyclonal affinity-purified ɑ-S5 and ɑ-AP41 antibodies, horseradish peroxidase-coupled anti-rabbit secondary antibodies (Thermo Scientific #31460) and Clarity Western ECL reagent (Biorad). Pictures were taken using a Chemidoc system (Biorad).

***Pyocin quantification by ELISA***

Microtiter 96-well plates (Corning #9018) were coated overnight with murine sera samples diluted 1/10 in PBS. After saturation with 2% bovine serum albumin (BSA, Sigma) in PBS and 4 washes using PBS + tween 20 0.05%, adherent S5 or AP41 were detected using specific primary antibodies (rabbit polyclonal affinity-purified ɑ-S5 or ɑ-AP41 antibodies, dilution 1/1000, 2 hours incubation) and biotin-SP-conjugated AffiniPure donkey anti-rabbit IgG (Jackson ImmunoResearch Laboratories 711-065-152, dilution 1/20000, 1 hour incubation), followed by incubation with peroxidase-conjugated streptavidin (Jackson ImmunoResearch Laboratories 016-030-084, 1µg/mL in PBS, 45 min incubation) with 4 washes between each step. After extensive washes, the plates were revealed using a citrate solution containing o-phenylenediamine dihydrochloride (OPD, Thermo Scientific 34006, 10 min incubation in the dark). The reaction was stopped using 2N H_2_SO_4_ and the plate read at OD 450 nm.

***Cytokine quantification***

Serum cytokines were quantified using the Bio-plex Pro Mouse Cytokine group 1 panel 8-plex kit (Biorad M60000007A). Samples were treated according to the manufacturer’s instructions and acquired on the Bio-Plex® 200 system (Biorad).

***Pyocin sensitivity assay***

100 µL of *P. aeruginosa* strain culture at OD_600_ = 0.6 was added to 10 mL of 0.8% sterile agar and poured on an LB agar plate. Unless otherwise stated, 5 µL of pyocin, organ homogenate or serum was spotted onto overlay plates and the plates incubated overnight at 37°C.

***In vitro killing assay in liquid cultures***

*P. aeruginosa* strains were grown until OD_600_ = 0.6, centrifuged and resuspended in the appropriate media (LB; LB + blood; LB + 2.2’ bipyridyl) at 108 CFU/mL. An inoculum was diluted and plated for CFU counts and pyocins at various concentrations were added at t=0. Samples were then incubated at 37°C and at each time point, 40 µL were taken out and incubated for 10 minutes at room temperature with trypsin (final concentration 100 µg/mL) before being serial-diluted and plated for CFU counts.
